# Progressive multifocal leucoencephalopathy in AIDS camouflaged with catatonia: A wolf in sheep's clothing

**DOI:** 10.4103/0019-5545.31625

**Published:** 2006

**Authors:** Pradeep Kumar, M.K. Jain

**Affiliations:** * Associate Professor, Department of Medicine, S.S. Medical College and Associated S.G.M. Hospital, Rewa, Madhya Pradesh; ** Professor and Head, Department of Medicine, S.S. Medical College and Associated S.G.M. Hospital, Rewa, Madhya Pradesh

**Keywords:** PML, AIDS, catatonia, akinetic mutism

## Abstract

Progressive multifocal leucoencephalopathy (PML) may pose a clinical and diagnostic dilemma. The patient may remain in a protracted psychotic state with negative symptoms, without overt features of dementia. The condition blends with catatonia, and eventually with akinetic mutism in a patient of AIDS in the absence of clinical evidence of an immunocompromised state. The present case report highlights the need for an in-depth clinical, biochemical and MRI assessment of patients with catatonia and akinetic mutism. Stupor of an ‘akinetic mutism’ pattern seems an important indication for HIV screening, particularly in high-risk patients.

## INTRODUCTION

Progressive multifocal leucoencephalopathy (PML) is a rare demyelinating disorder caused by the opportunistic JC papovavirus. It is an occasional complication of chronic diseases affecting the reticulo-endothelial system such as lymphoma, Hodgkin disease, leukaemia, sarcoidosis, tuberculosis; patients undergoing immunosuppressive therapy in malignancy and post-organ transplantation, and in AIDS. HIV-associated cases account for up to 85% of all cases of PML^1^ and there is a likelihood of further increase with the rising trend in AIDS. A case of PML associated with AIDS is described with clinical features of psychoticism with negative features leading to catatonia and eventually akinetic mutism without clinical evidence of an immunocompromised state.

## THE CASE

A 32-year-old married male, graduate, with stable premorbid personality traits, without any genetic loading, non-addict, normotensive, non-diabetic developed aberrant behaviour for the first time. This led to irregularities and inattentiveness at work, increasing global withdrawal, emotional aloofness, irritable and impulsive outbursts, neglect of personal hygiene and personal care, delusion of reference, fragmentary persecutory delusion, impaired judgement and insight for illness. The altered behaviour persisted for more than 6 months when he suddenly developed profound psychomotor retardation, inability to speak, tremulousness while walking and held anything that he came in contact with. He did not communicate any depressive symptom. His condition progressed to complete immobility with abnormal postures and mutism, and he needed assisted feeding. Response to strong noxious stimuli were absent although spontaneous movements were present. Although he slept most of the time, he nevertheless looked alert with his eyes chasing objects during the waking phase.

There was no history of fever, lymphadenopathy or diarrhoea. No history of head injury, seizure or ear discharge was reported.

He was diagnosed as a case of functional catatonia and was referred to our centre.

General examination was within normal limits. There was no cutaneous lesion, oral thrush, hepatosplenomegaly or lymphadenopathy. Pulmonary tuberculosis was not evident both on clinical and radiological examination.

**Fig 1:**
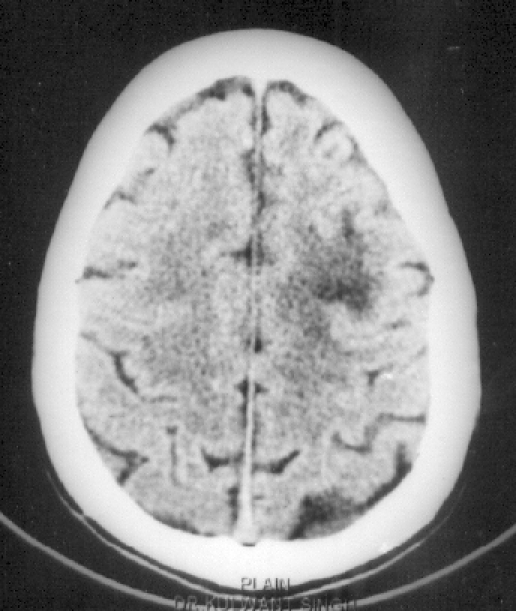
CT scan brain showing a non-enhancing hypodense lesion in the left frontal cortex with sulci prominent in relation to the age.

Higher function examination revealed an extreme degree of hypokinesia, mutism and catalepsy for more than 30 seconds. He looked conscious, vigilant with positive eye-tracking movements, and loss of apparent condition-avoidance response. Orientation, attention, concentration, memory and judgement could not be commented upon due to mutism. His affect was blunt without weeping or laughing spells (no pseudobulbar pattern). Cerebellar signs could not be tested; mild motor ataxia was present. There was no sensory–motor deficit but bladder and bowel incontinence was present. A syndromic diagnosis of catatonia was made. The patient was subjected to a battery of investigations. Routine blood and urine examination, hepatic and renal function tests were within normal limits except mild iron deficiency anaemia; serum B_12_ and HbAIC levels were within the normal range. The optic fundus was normal. CSF pressure and biochemical parameters were normal and the VDRL test was negative. X-ray chest and USG abdomen were normal. CT scan revealed a single non-enhancing hypodense lesion in the left frontal cortex and mild cerebral atrophy for his age (Fig 1).

**Fig 2:**
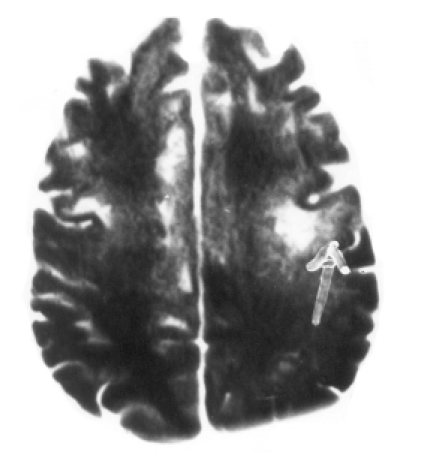
T_1_W axial image at the level of the centrum semi-ovale. Multiple focal hypointense lesions are seen bilaterally in the subcortical white matter of the frontal lobe causing widening of the overlying gyri on the left side (arrow).

**Fig 3:**
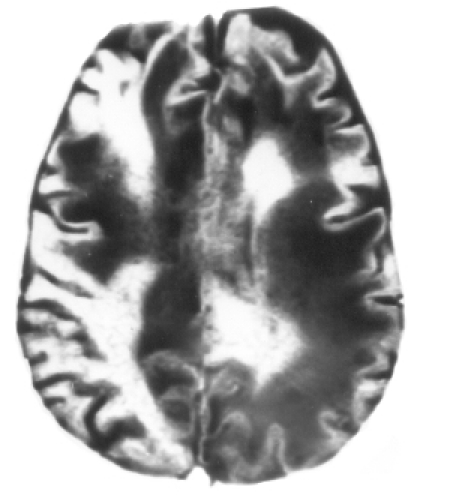
T_2_W axial image at the level of the centrum semi-ovale. Confluent and discrete T_2_ hyperintense lesions are seen in the subcortical white matter with the involvement of ‘u’ fibres bilaterally in an asymmetrical distribution.

**Fig 4:**
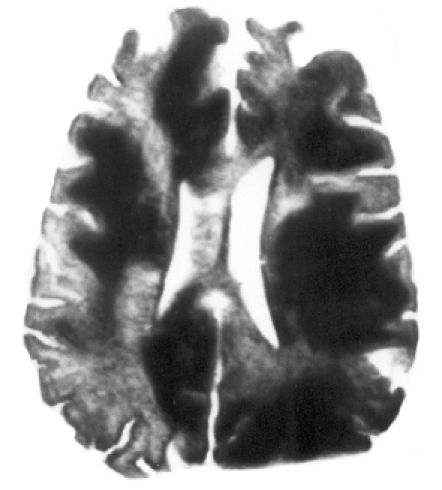
FLAIR axial image at the level of the body of the lateral ventricle. Confluent hyperintense lesions are seen in the subcortical white matter with involvement of ‘u’ fibres bilaterally in an asymmetrical distribution.

**Fig 5:**
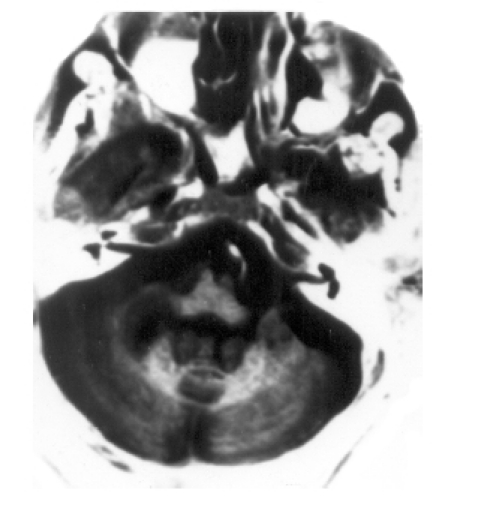
T_2_W axial image at the level of the medulla. T_2_ hyperintense lesions are seen in the cerebellar white matter.

After 7 days of hospitalization, the patient revealed positive grasp, rooting and glabellar tap reflexes, and right hemiparesis (grade III). At that point, considering the focal cerebral damage, he was categorized as a case of akinetic mutism. The patient was found to be positive for HIV-1 and -2, which was confirmed by the Western blot test. The total leucocyte count was 4990 cells/cmm, lymphocytes were 16%, the absolute lymphocyte count was 800 cells/cmm. The absolute CD3+ T lymphocyte count was 528 cells/cmm. The absolute CD4+ T helper cell count was 339 cells/cmm (normal range 337–1690 cells/cmm). Analysis was done using the flowcytometry method. MRI, done after 5 days of CT scan, revealed multiple, confluent, non-enhancing, discrete, asymmetric, subcortical white matter lesions, maximum in the frontoparieto-occipital and cerebellar regions without mass effect (Figs 2–5). Ante-mortem brain biopsy was not feasible.

A final diagnosis of PML in AIDS was made for inducing the state of akinetic mutism.

## DISCUSSION

Catatonia as a clinical syndrome is an elusive entity. PML in AIDS which presents exclusively as catatonia raises crucial concerns about diagnostic conceptualization and serious lapses in investigation of such patients, as well as risk management of caregivers and hospital staff.

HIV infection in this patient presented solely as PML with no identifiable clinical evidence of immunosuppression. The CD4+ T helper cell count of 339 cells/cmm (normal range 337–1690 cells/cmm) further confirms relatively good immunological competence. Only 6% of patients with PML without immunocompromised status have been reported. However, these were all anecdotal reports.^2^ There is conflicting evidence as to whether JC viral DNA is present within the brain of non-immunocompromised patients.^1^ However, the development of so-called ‘primary’ PML in patients without immunosuppression would suggest that the virus may lie dormant within the nervous system.^1^ Irrespective of the immune status, the JC virus remains latent in the kidney and may be detected in the urine. Whether PML results from reactivation of latent virus in the brain or due to haematogenous spread is yet unclear. Pathological findings in favour of haematogenous spread point towards the predilection of PML for the grey/white interface around the end arterioles.^1^ The present case report highlights the need for further exploration to unravel this yet unresolved issue.

PML that presents as a psychotic state with predominantly negative symptoms which develop in a subacute manner, and run a protracted course, ultimately blend with catatonia in the absence of other neurological signs such as overt dementia, motor function abnormalities, cranial nerve palsies may well camouflaged with functional catatonia. Catatonia, the term used by Karl Kahlbaum, 1863^3^ is often an intricate neurobehavioural syndrome with diverse primary functional (affective disorder, schizophrenia) and secondary organic causes. It is frequently encountered in general medical and psychiatric wards. The index case also had a triad of mutism, akinesia or severe hypokinesia and catalepsy which fulfilled the criteria of catatonia.

It was the late onset emergence of the frontal grasp, rooting and glabellar tap reflexes which pointed towards underlying focal cerebral damage and enabled us to precisely predict the impending right hemiplegia and double incontinence that developed in 3 weeks. The term ‘akinetic mutism’ as coined by Cairns *et al*.^4^ has two crucial diagnostic factors^5^—first, a seeming wakefulness without recognizable content and, second, a relative paucity of signs implying significant damage to the descending motor pathways, which explain the immobile state. The present case had immobility and mutism, which were not based on a primary motor disturbance but the evidence of focal cerebral damage allowed it to be classified as akinetic mutism and more precisely as ‘frontal-akinetic mutism’.^6^ The possibility of ‘locked-in syndrome’ was ruled out as the majority of the descending motor pathways to the spinal cord and lower cranial nerve motor nuclei were intact, and mutism was associated with spontaneous motor move-ments. The condition therefore fulfils the criteria for the syndromic diagnosis of catatonia followed by akinetic mutism. Catatonia in this case may represent early neurotransmitter dysfunction, affecting areas where structural damage could induce akinetic mutism. Core cognitive deficits in negative schizophrenia, particularly in the area of executive function and working memory, have been emphasized by recent investigators.^7^ Hairline differences among various clinical syndromes of functional and/or organic CNS origin involving the fronto-limbic-striatal circuitry^8^ as a common pathway could easily be missed during cursory physical and neuro-psychiatric evaluation, and may be of great clinical relevance in a sensitive disease such as AIDS.

MRI facilities in India are few, especially in government medical colleges. It seems the mainstay of diagnosis of PML as antemortem brain biopsy is not feasible. The cost of MRI compared with CT scan is yet another limiting factor for the diagnosis of such cases. The deceptive role of CT scan in diagnosing such demyelinating disorders is obvious (Fig 1). Delayed identification of such cases of PML in AIDS not only deprives them of prompt management with antiretroviral therapy but also poses a risk to caregivers, including therapists. It is strongly recommended that all patients presenting with catatonia/akinetic mutism, particularly those belonging to a high-risk group for AIDS, must be routinely screened for HIV and subjected to MRI of the brain.
